# Backstepping Controller for Nanopositioning in Piezoelectric Actuators with ANN Hysteresis Compensation

**DOI:** 10.3390/mi16040469

**Published:** 2025-04-15

**Authors:** Asier del Rio, Oscar Barambones, Eneko Artetxe, Jokin Uralde, Isidro Calvo

**Affiliations:** Department of Systems Engineering and Automatic Control, Faculty of Engineering of Vitoria-Gasteiz, University of the Basque Country (UPV/EHU), 01006 Vitoria-Gasteiz, Spain; eneko.artetxe@ehu.eus (E.A.); jokin.uralde@ehu.eus (J.U.); isidro.calvo@ehu.eus (I.C.)

**Keywords:** piezoelectric actuator, backstepping controller, artificial neural network, hysteresis compensation, intelligent control

## Abstract

Piezoelectric actuators (PEAs) are widely used in high-precision applications but suffer from nonlinear hysteresis effects that degrade positioning accuracy. To address this challenge, this study presents a backstepping controller with an Artificial Neural Network (ANN)-based feedforward compensation scheme to enhance trajectory tracking performance. The ANN compensates for the hysteresis effects, while the backstepping strategy ensures robust reference tracking. The proposed controller is validated through real-time experiments using a piezoelectric actuator system. Comparative analysis with a conventional PID controller demonstrates the superiority of the backstepping approach, achieving significantly lower tracking errors across different reference signals and frequencies. Error metrics have been employed to confirm the improved accuracy and robustness of the proposed method. These findings highlight the effectiveness of the proposed ANN-enhanced backstepping control in overcoming hysteresis-related challenges in precision positioning applications.

## 1. Introduction

Piezoelectric actuators (PEAs) are widely used in various industrial applications due to their suitability as microactuators [1]. They have been established as ad hoc solutions for problems requiring high-precision nanopositioning in mechanical systems that demand fast response and robustness, ranging from aerospace applications [2] to semiconductor manufacturing [3]. Other typical applications include the positioning of lenses [4], mirrors [5], diaphragms [6], and other optical components in cameras [7], telescopes [8], microscopes [9], lasers [10], and image stabilization systems [11].

Their main advantages include high-frequency response, exceptional robustness, and high resolution [12]. These systems can deliver substantial forces and displacements with minimal power consumption, making them well suited for applications that require both high dynamic performance and energy efficiency [13].

However, the inherent hysteresis of PEAs significantly affects their positioning accuracy [14]. This phenomenon arises from the properties of the materials used in their construction and is caused by the irreversible alteration of their internal structure when exposed to electric fields, leading to a delay or memory effect [15]. Consequently, accurately modeling and compensating for these effects is imperative for achieving high-precision control with PEAs [16].

Hysteresis modeling techniques for PEAs, such as the Jiles–Atherton (J–A) [17], Bouc–Wen [18], Preisach [19], Prandtl–Ishlinskii (P–I) [20,21,22], Krasnosel’skii–Pokrovskii [23], Duhem [24], LuGre [25], Dahl [26], and Maxwell-slip [27] models, are widely used due to their ability to capture the nonlinear and memory-dependent behavior of these actuators. These models vary in complexity and computational cost, with some offering phenomenological descriptions (e.g., Preisach and P–I models) [28] and others providing physics-based formulations (e.g., J–A and LuGre models) [29].

While these approaches can achieve accurate hysteresis representation, they often require extensive parameter identification and may struggle with model generalization when operating conditions change [30]. In contrast, Artificial Neural Networks (ANNs) offer a data-driven alternative that can learn complex hysteresis characteristics without explicitly defining mathematical structures [31]. This is due to the fact that ANNs are universal approximators capable of modeling any correlation between arbitrary input data with their corresponding outputs [32]. ANNs provide enhanced adaptability and generalization capabilities, reducing the need for extensive system identification. Moreover, they can efficiently approximate nonlinear mappings with high accuracy, making them particularly advantageous in real-time control applications where traditional models may become computationally expensive or less effective under varying conditions, as in this case with the strong nonlinear dynamics of the PEA’s hysteresis effects. Given the limitations previously discussed regarding hysteresis models, in this work the hysteresis compensation in the controller was incorporated using an ANN.

This study presents a backstepping controller with ANN-based feedforward hysteresis compensation for precise PEA positioning. The use of an ANN addresses the challenge of modeling the complex nonlinear dynamics of PEAs. To validate the proposed solution, the controller is implemented in a real-time setup. The performance of the backstepping controller is compared against that of a PID controller as a benchmark of an industry-standard controller.

The paper is structured as follows: Section 2 provides a detailed overview of the hardware and experimental setups employed, as well as the process conducted for the identification of the hysteresis effects and the training of the ANN for feedforward compensation. Section 3 includes the design of the tested controllers and a stability proof of the proposed solution. Section 4 presents the results of the experiments and a graphical and numerical analysis. Finally, Section 5 provides a summary that outlines the main findings and accomplishments of this research.

## 2. Materials and Methods

### 2.1. Hardware Description

The PK4FYC2 is a PEA composed of a discrete stack configuration, integrating four metal foil strain gauges arranged in a full-bridge Wheatstone circuit. These strain gauges are affixed to a robust epoxy resin coating that encapsulates both the actuator and its electrical leads, ensuring protection and durability. Each strain gauge is further shielded by a short strip of polyimide tape, which helps preserve their functionality while they monitor the actuator’s displacement. The actuator itself is constructed from multiple piezoelectric chips, which are securely bonded together using a combination of epoxy resin and glass beads. This design enables it to achieve a maximum displacement of 38.5 μm with a tolerance of ±15%, according to the manufacturer. Each strain gauge exhibits a resistance of 350 Ω and a gauge factor of 2. Additional technical specifications are provided in Table 1.

The KPZ101 Piezo Controller is a compact, single-channel control and drive unit designed for both manual and automated operation of various piezoelectric stacks and actuators. Capable of supplying a drive voltage of up to 150 V with a maximum current of 7.5 mA, this controller supports operating bandwidths of up to 1 kHz. It is fully compatible with the PK4FYC2 Piezoelectric Stack and, when used alongside the KSG101 Strain Gauge Reader, enables high-precision closed-loop control.

Additionally, the KPZ101 features a digitally encoded adjustment potentiometer, allowing for precise manual positioning of the piezo actuator, with an integrated voltage display for real-time monitoring. High-voltage output can be directly controlled via the low-voltage input connector, while a dedicated low-voltage output provides convenient access for monitoring the high-voltage output. The technical specifications for both KPZ101 Piezo Controller and KSG101 Strain Gauge reader, as well as the specifications of the AMP002 Pre-Amplifier required by the reader, are displayed in Table 2. Note that this equipment is manufactured by Thorlabs, Inc. and sourced from Munich, Germany.

Both the actuation and measurement signals are managed by a dSpace DS1104 board, which features real-time interface (RTI) capabilities. This hardware minimizes compilation time for driving algorithms while enabling real-time control tuning. The DS1104 board is connected to a computer via a PCI bus and operates in conjunction with dSpace ControlDesk 2022-B software, facilitating real-time control, data visualization, and acquisition.

**Figure 1:**
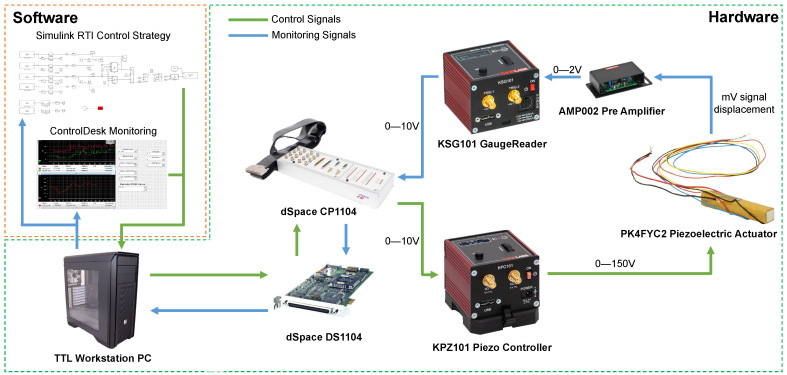
Hardware–software workflow and interconnections.

The control architecture is implemented in Simulink 2022b and embedded into the DS1104 board using dSpace RTI. A sampling rate of 1 kHz is set for all experiments, ensuring synchronization between data acquisition and the hardware’s physical constraints. A schematic representation of all hardware–software interconnections is provided in Figure 1.

### 2.2. Hysteresis Analysis and ANN Modeling

Hysteresis in PEAs is a phenomenon in which the actuator’s displacement is influenced not only by the current input voltage but also by its past variations. This effect stems from the intrinsic properties of the material used in PEAs, which naturally exhibit hysteretic behavior. The underlying cause of this phenomenon is the irreversible alterations in the internal structure of the material when exposed to an electric field, leading to a lag or memory effect in the actuator’s response.

**Figure 2:**
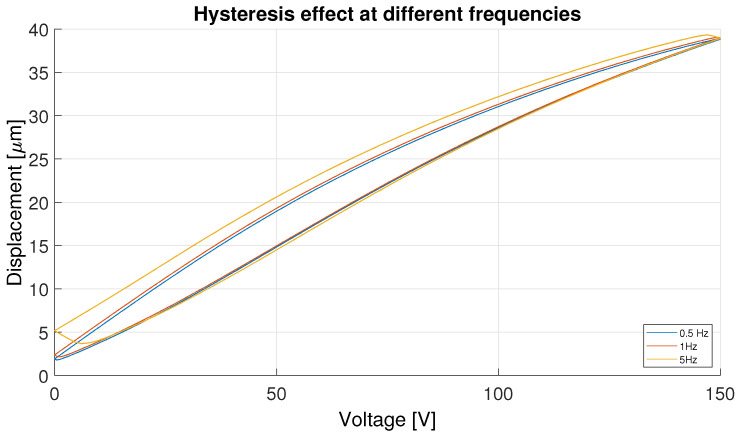
Hysteresis effect with different frequencies.

As a result, when the input voltage is adjusted, either increasing or decreasing, the displacement of the actuator does not immediately follow the applied signal. Instead, its response may deviate from an ideal trajectory, depending on the previous voltage history. This nonlinearity poses challenges in controlling PEAs, as it introduces memory-dependent behavior that must be addressed within the control algorithm. Figure 2 shows this effect, depicting the different hysteresis curves with different frequencies employing triangular signals of 0–15V to showcase the effect described above. Consequently, accurately characterizing and modeling hysteresis is crucial for developing control strategies capable of predicting and compensating for these effects, ensuring precise actuator performance.

To tackle these challenges, ANNs have emerged as a promising alternative. ANNs excel at capturing complex non-linear dynamics directly from data, making them particularly effective for modeling systems that exhibit hysteresis and other non-linear behaviors. By incorporating an ANN into the control scheme, it becomes possible to effectively compensate for both linearity deviations and hysteresis effects, thereby improving accuracy and robustness.

**Figure 3:**
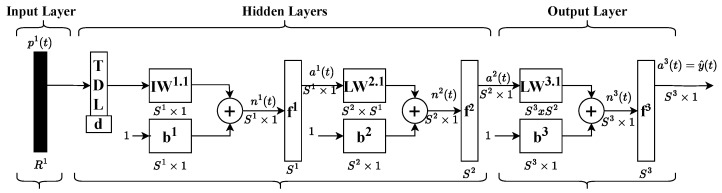
TDNN architecture.

For this purpose, a Time Delay Neural Network (TDNN) was selected. Unlike conventional feedforward networks, TDNNs incorporate a tap delay line within the input weights, enabling the network to retain a finite dynamic response to sequential input data. This characteristic is essential for modeling hysteresis systems, as past inputs significantly influence the system’s output. A diagram illustrating the TDNN architecture is provided below in Figure 3.

The training process began with data collection, where multiple cycles of triangular and sinusoidal signals with varying frequencies and amplitudes were gathered. This dataset underwent preprocessing and curation to ensure high-quality inputs. Subsequently, the TDNN architecture was designed by determining the number of layers, neurons per layer, and activation functions. The network was initialized with random weights and trained using a dataset split into training, validation, and testing subsets. The backpropagation algorithm was employed to iteratively adjust the weights and optimize model performance. If the training results did not meet the expected performance criteria, architectural modifications, such as altering the number of layers, neurons, or activation functions, were made, and the training process was repeated.

Once satisfactory results were achieved, the model’s generalization ability was validated using additional datasets. The final network, selected for its optimal performance based on the RMSE criterion, consists of an input layer, a hidden layer composed of 10 neurons, and a regression output layer responsible for predicting hysteresis displacement. Further details on the training outcomes are presented in Table 3.

## 3. Control Strategy Design

Since hysteresis is an inherent effect that cannot be entirely eliminated, a dedicated control strategy is designed and implemented to minimize position errors, enabling the use of the PEA in high-precision applications. To achieve optimal tracking accuracy, state observers or a combination of feedforward (FF) and feedback control are commonly employed.

In this work, an ANN is utilized for system identification, a technique widely applied in clustering, pattern recognition, classification, optimization, and prediction tasks. Specifically, a Time Delay Neural Network (TDNN) is implemented for predictive modeling, where input signals are incorporated with specified time delays to improve prediction accuracy.

**Figure 4:**
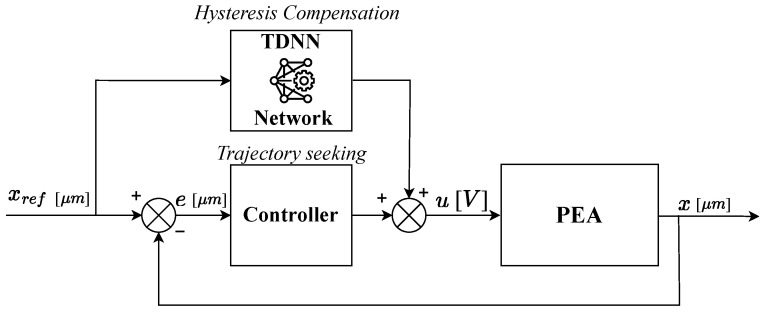
Control scheme.

The ANN is responsible for compensating hysteresis effects. However, to ensure robust performance, a fully integrated control architecture must include a feedback controller to correct errors during trajectory tracking. This necessity leads to a hybrid approach that combines a feedback loop for trajectory regulation with a feedforward compensation mechanism, as illustrated in Figure 4. The closed-loop controller is designed to generate precise input signals, ensuring both fast and accurate reference tracking while minimizing errors.

For this purpose, a backstepping controller is proposed and evaluated against a standard PID controller, which serves as a benchmark for performance comparison.

### 3.1. PID Controller

The Proportional-Integral-Derivative (PID) controller is a fundamental control algorithm extensively applied in industrial settings. Its widespread adoption is attributed to its intuitive operation, straightforward design, and well-established theoretical foundation. Due to its prevalence in both industry and academia, it is frequently used as a benchmark for evaluating the performance of more advanced control strategies, as demonstrated throughout this study.

In this work, the PID controller was implemented based on Equation (1), incorporating an anti-windup mechanism to mitigate the effects of integral action saturation caused by control signal constraints.

(1) $$u\left(t\right)=sat\left(K_{p}e\left(t\right)+K_{i}\int_{t_{0}}^{t}e\left(t\right)dt+b\left(t\right)+K_{d}\frac{d}{dt}e\left(t\right)\right)$$

Here, u(t) represents the controller’s output signal, while e(t) denotes the error signal. The parameters $K_{p}$ and $K_{i}$ correspond to the proportional and integral gain coefficients, respectively. The function sat() imposes a constraint on the controller output, limiting it within the 0–150 V range. Additionally, b(t) is the back-calculation term, which is determined by integrating the saturation error over the duration of the saturation period, as defined in Equation (2).

(2) $$b\left(t\right)=K_{b}\int_{t_{start}}^{t}e\left(t\right)dt$$

The controller parameters were determined through an iterative experimental process aimed at minimizing the Integral of Absolute Error (IAE). The final parameter values obtained from this optimization procedure are summarized in Table 4.

### 3.2. Backstepping Controller

This section covers the design of the backstepping controller for reference tracking of the PEA. First, the model used for the dynamics of the PEA is described in Equation (3), where *x* represents the displacement of the actuator over time, *m* represents the mass, *b* is the damping coefficient, *k* is the spring constant, *d* is the piezoelectric coefficient, *u* is the voltage applied to the actuator, and *h* accounts for hysteresis effects.

(3) $$m\ddot{x}+b\dot{x}+kx=k\left(du-h\right)$$

The system described by Equation (3) can be rearranged as follows to obtain a strict-feedback system ruled by two subsystems with *x* and $\dot{x}$ governing the dynamics

(4) $$\ddot{x}=-\frac{b}{m}\dot{x}-\frac{k}{m}x-\frac{k}{m}h+\frac{kd}{m}u$$

The first subsystem is designed to force *x* to track the reference signal $x_{ref}$, defining error $e_{1}$ as

(5) $$e_{1}=x-x_{ref}$$

with the derivative ${\dot{e}}_{1}$ of the $e_{1}$ error being

(6) $${\dot{e}}_{1}=\dot{x}-{\dot{x}}_{ref}$$

A Lyapunov candidate function for the first subsystem is selected as

(7) $$V_{1}=\frac{1}{2}{e_{1}}^{2}$$

An auxiliary variable *v* is introduced to facilitate the control design

(8) $$v={\dot{x}}_{ref}-k_{1}e_{1}-e_{2}$$

where $k_{1}$ is an arbitrary positive constant and $e_{2}$ the error tracking of the second subsystem defined as

(9) $$e_{2}=\dot{x}-v=\dot{x}-{\dot{x}}_{ref}+k_{1}e_{1}+e_{2}$$

which can be simplified as

(10) $$\dot{x}-{\dot{x}}_{ref}=-k_{1}e_{1}$$

Thus, the derivative of the first Lyapunov function ${\dot{V}}_{1}$ would be

(11) $$\dot{V}=e_{1}{\dot{e}}_{1}$$

Substituting Equation (6) into (10) and subsequently into (11), yields

(12) $$\dot{V}=-k_{1}{e_{1}}^{2}$$

Since $k_{1}>0$, the stability of the first subsystem is guaranteed. Continuing with the second subsystem, the augmented Lyapunov function is defined as

(13) $$V_{2}=V_{1}+\frac{1}{2}{e_{2}}^{2}$$

Differentiating $V_{2}$ with respect to time gives

(14) $${\dot{V}}_{2}={\dot{V}}_{1}+e_{2}{\dot{e}}_{2}=-k_{1}{e_{1}}^{2}+e_{2}\left({\dot{x}}_{2}-\dot{v}\right)$$

Substituting Equation (4) in (14) results in

(15) $${\dot{V}}_{2}=-k_{1}{e_{1}}^{2}+e_{2}\left(-\frac{b}{m}\dot{x}-\frac{k}{m}x-\frac{kh}{m}+\frac{kd}{m}u+D-\dot{v}\right)$$

where *D* represents the model parameter inaccuracies. Now, the control law described by Equation (16) is implemented, where an commuting variable is added to compensate the model inaccuracies represented by *D*, where β and $k_{1}$ are positive constant design parameters.

(16) $$u=\frac{m}{kd}\left(\frac{b}{m}\dot{x}+\frac{k}{m}x+\frac{k}{m}h-\dot{v}-k_{2}e_{2}-\beta sign\left(e_{2}\right)\right)$$

Implementing this control law into Equation (15) gives

(17) $${\dot{V}}_{2}=-k_{1}{e_{1}}^{2}-k_{2}{e_{2}}^{2}+De_{2}-\beta \left|e_{2}\right|$$

Thus, if β≥|D|, then ${\dot{V}}_{2}\leq 0$ ensuring the stability of the overall closed-loop system. The parameter values of the backstepping controller employed in the experiment are shown in Table 5.

## 4. Results

Building upon the design process outlined in previous sections, a series of experiments were conducted on a real-time control platform to evaluate the effectiveness of the proposed controllers. These experiments aimed to compare the performance of the backstepping controller against that of the PID controller by tracking both triangular and sinusoidal reference signals at varying frequencies, thereby assessing their response under different operating conditions.

**Figure 5:**
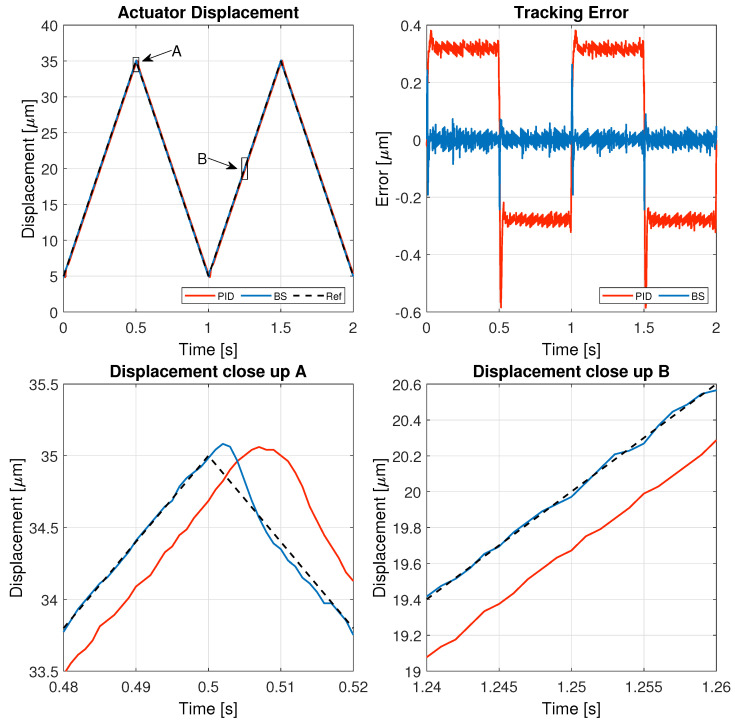
Results of the experiment with 1 Hz triangular reference signal.

**Figure 6:**
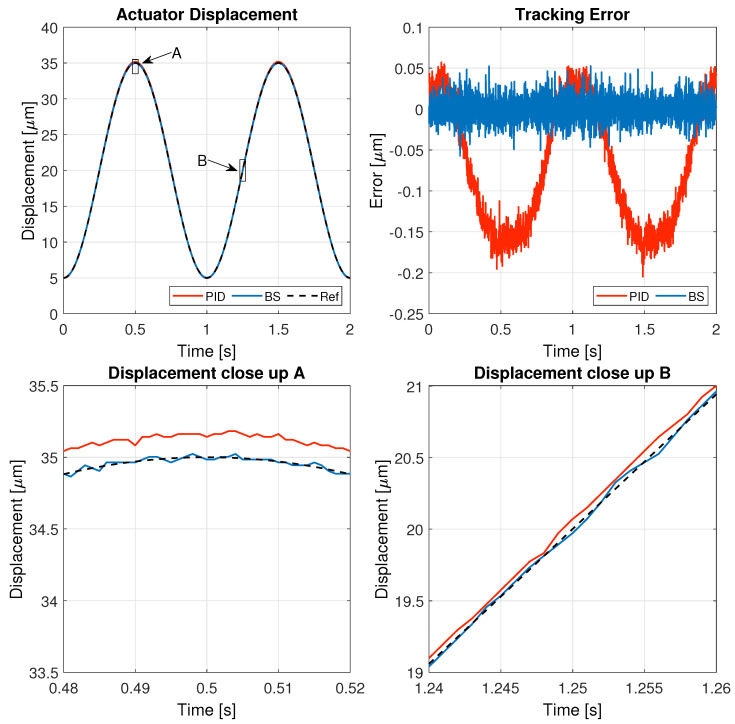
Results of the experiment with 1 Hz sinusoidal reference signal.

**Figure 7:**
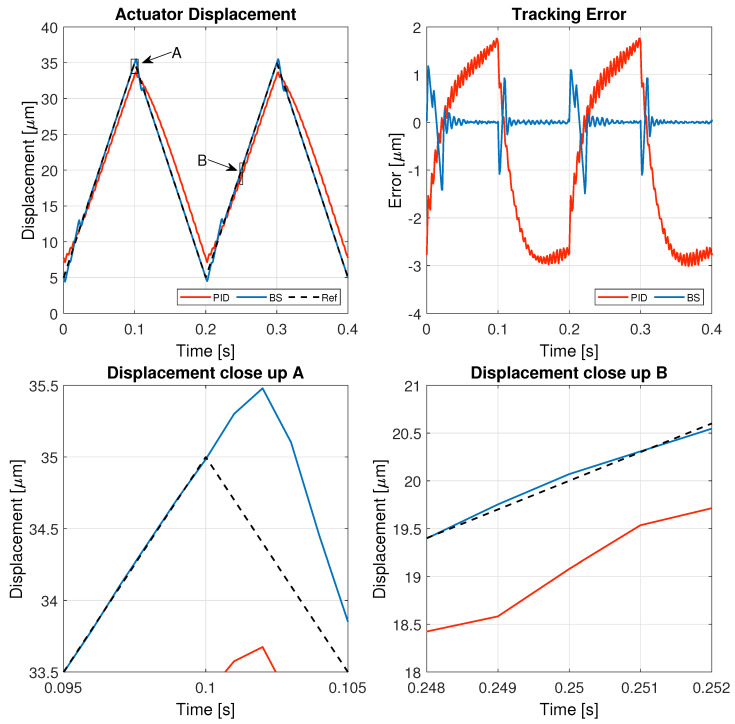
Results of the experiment with 5 Hz triangular reference signal.

**Figure 8:**
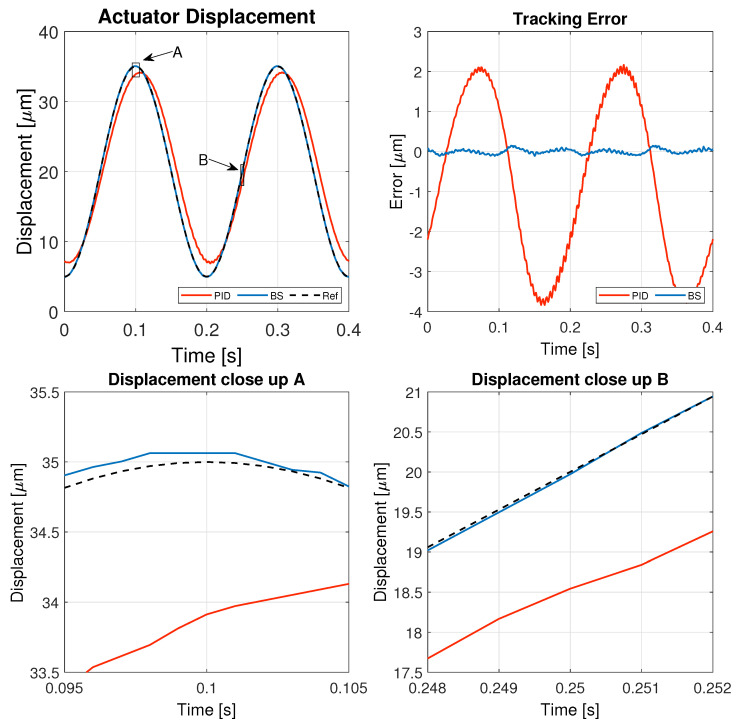
Results of the experiment with 5 Hz sinusoidal reference signal.

The results of these tests are detailed in this section, specifically illustrating the system’s behavior for a 1 Hz triangular signal in Figure 5, a 1 Hz sinusoidal signal in Figure 6, a 5 Hz triangular signal in Figure 7, and a 5 Hz sinusoidal signal in Figure 8.

Beyond graphical analysis, and in line with the objective of accurately tracking a reference trajectory, three widely used error metrics in control system evaluation were employed. Specifically, the Integral Absolute Error (IAE), Root Mean Square Error (RMSE), and Relative Root Mean Square Error (RRMSE) were selected as quantitative performance indicators. These metrics, defined in Equation (18), provide an objective basis for comparing the effectiveness of the proposed control strategies against conventional approaches.

(18) $$\begin{array}{c} IAE=\frac{1}{n}\sum_{i=1}^{n}\left(\hat{y_{i}}-y_{i}\right)\;\\ RMSE=\sqrt{\frac{1}{n}\sum_{i=1}^{n}{\left(\hat{y_{i}}-y_{i}\right)}^{2}}\;\\ RRMSE=\sqrt{\frac{\frac{1}{n}\sum_{i=1}^{n}{\left(\hat{y_{i}}-y_{i}\right)}^{2}}{\sum_{i=1}^{n}\hat{y_{i}}}}\cdot 100 \end{array}$$

where *n* denotes the total number of samples, $\hat{y_{i}}$ represents the reference value at the *i*-th sample, and $y_{i}$ corresponds to the actual system output at the same instance.

Additionally, to account for the effort exerted by the controllers, a metric called IAU (Integral of Absolute Voltage Effort) is applied, which is represented in Equation (19) as follows:

(19) $$IAU=\frac{1}{n}\sum_{i=1}^{n}\left|u_{i}\right|$$

where $u_{i}$ represents the applied voltage value at the *i*-th sample.

Figure 5, Figure 6, Figure 7 and Figure 8 illustrate the test results used to assess the performance of the controller implemented in this study, while the corresponding performance error metrics are summarized in Table 6.

The results demonstrate that both controllers can track the reference signal effectively. However, the backstepping controller significantly improves tracking accuracy, achieving a lower steady-state error and a smoother response. In contrast, the PID controller exhibits larger deviations, particularly during sharp transitions of the triangular signal. The close-up view of the displacement further confirms that the backstepping approach ensures a more precise trajectory, reducing overshoot and oscillations, which highlights its superiority in handling reference signals with sudden variations.

When tracking a low-frequency sinusoidal reference, both controllers maintain good performance, but the backstepping controller consistently achieves a lower tracking error. The PID controller shows small but noticeable deviations, especially near peaks and valleys of the sinusoidal wave. The close-up displacement view reveals that the backstepping controller follows the reference more accurately, with reduced phase lag and smoother transitions. These results confirm that backstepping provides a more precise and stable response, enhancing system reliability for applications requiring high precision.

At a higher frequency, the limitations of the PID controller become more evident as it struggles to maintain accurate tracking, resulting in a higher tracking error and delayed response. In contrast, the backstepping controller adapts better to the rapid changes in the reference signal, demonstrating improved robustness against high-frequency variations. The close-up views of the displacement indicate that backstepping effectively follows the sharp transitions of the triangular wave with minimal delay and error, further validating its superiority in handling demanding dynamic conditions.

The increased frequency of the sinusoidal input poses a greater challenge for both controllers, leading to an overall increase in tracking error. However, the backstepping controller still outperforms the PID controller, exhibiting a significantly smaller error and a more stable response. The close-up analysis confirms that backstepping provides better phase alignment with the reference signal, while the PID controller introduces noticeable lag and oscillations. These findings highlight the backstepping controller as a more effective solution for high-frequency applications, where precision and rapid adaptation are critical.

Al the results from the error metric analysis are gathered in Table 6.

The numerical analysis of the experiments using the IAE metric confirms the superior performance of the ANN-backstepping controller over the traditional PID controller. Across all tested reference signals, the backstepping approach achieves significantly lower error values, demonstrating its enhanced tracking accuracy. The most remarkable improvement is observed for the 1 Hz triangular reference, where the IAE is reduced from 2.3207 (PID) to 0.034315 (backstepping), representing an improvement factor of over 67 times. Similarly, for the 1 Hz sinusoidal signal, the error is reduced by approximately 40 times, while for the 5 Hz sinusoidal case, the backstepping controller achieves an error reduction of over 40 times as well. Even in the most challenging scenario of 5 Hz triangular tracking, where high-frequency changes make accurate tracking more difficult, backstepping still outperforms PID with a significant reduction in error.

Looking into the IAU metric that measures the controller effort, it is observed that the control action of the proposed controller is slightly smoother, exhibiting a lower average voltage due to the greater progressiveness of the control action when comparing to the benchmark PID.

These results clearly demonstrate that the ANN-backstepping controller provides a substantial improvement in trajectory tracking, minimizing deviations and ensuring better overall system performance.

## 5. Conclusions

This study introduced a backstepping controller with ANN-based hysteresis compensation for precise trajectory tracking in piezoelectric actuators (PEAs). The hysteresis effect, which significantly impacts positioning accuracy, was modeled using a Time Delay Neural Network (TDNN). This network was trained with experimentally collected data to predict and compensate for hysteresis nonlinearity, improving system performance without requiring complex mathematical hysteresis models.

The experimental validation demonstrated that the proposed controller significantly outperforms the conventional PID approach in terms of tracking accuracy, particularly in scenarios involving abrupt reference changes and high-frequency signals.

The experimental results confirmed that the backstepping controller achieves a lower steady-state error, smoother transitions, and reduced overshoot compared with the PID controller. While both controllers exhibited effective tracking performance at low frequencies, the backstepping approach consistently maintained a lower tracking error, reduced phase lag, and improved stability. At higher frequencies, the superiority of the backstepping controller became even more pronounced, as it demonstrated enhanced robustness in handling rapid reference variations and minimized response delays. The numerical analysis further supported these findings, with the IAE metric indicating a remarkable reduction in error across all tested reference signals. Notably, the backstepping controller achieved over 67 times improvement in error reduction for a 1 Hz triangular reference and approximately 40 times improvement for sinusoidal signals.

## Figures and Tables

**Table 1:** PK4FYC2 Piezoelectric stack specifications.

Specification	Value
Drive Voltage Range	0 to 150 V
Displacement at 150 V (No Load)	38.5 µm ± 15%
Hysteresis	<15%
Load for Maximum Displacement	400 N
Recommended Preload	<400 N
Blocking Force at 150 V	1000 N
Resonant Frequency (No Load)	34 kHz ± 15%
Anti-Resonant Frequency (No Load)	42 kHz ± 15%
Impedance at Resonant Frequency	150 mΩ
Dissipation Factor (1 kHz, 1 $V_{RMS}$)	<2.0%
Capacitance (1 kHz, 1 $V_{RMS}$)	3.5 µF ± 15%
Operating Temperature	−25 to 65 °C
Curie Temperature	230 °C
Bridge Arm Resistance	350 Ω ± 0.3%
Gauge Factor	2
Maximum Recommended Excitation Voltage	4.5 $V_{RMS}$
Dimensions	Width: 7.3 mm Maximum
	Height: 6.5 mm Maximum
	Length: 36.0 mm ± 0.1 mm

**Table 2:** Technical specifications of KPZ101 Piezo controller, KSG101 Strain Gauge reader, and AMP002 Pre-Amplifier.

**KPZ101 driver**	**Value**	**Units**
Output driving Voltage Range	0–150	V
Input Reference Voltage Range	0–10	V
Maximum Output Bandwidth	1	kHz
**KSG101 reader**	**Value**	**Units**
Output Range	0–10	V
Measurement Resolution	1	nm
**AMP002 Pre-Amplifier**	**Value**	**Units**
Input Supply	±15	V
Output Range	0–2	V

**Table 3:** TDNN training results.

Parameters	Values
Neurons	10, 1
Delay Sequence Length	25
Activation Fuctions	Tanh, Purelin
Training Function	Levenberg-Marquardt
Epoch	10,000
Initial μ	0.001
μ Decay factor	0.1
μ Increase Factor	10
Perform Function	MSE
Best Training Performance	6.9194 $\times \;10^{-7}$
Best Validation Performance	7.1279 $\times \;10^{-7}$
Best Test Performance	7.1125 $\times \;10^{-7}$

**Table 4:** PID parameters.

Parameters	$K_{p}$	$K_{i}$	$K_{d}$	$K_{b}$
Values	0.5	1200	0	100

**Table 5:** Backstepping controller parameters.

Parameters	$K_{1}$	$K_{2}$	β
Values	500	30,000	1000

**Table 6:** Error comparison between controllers with different reference signals.

Reference	IAE		
	PID	ANN-BackStepping	Difference
Triangular 1 Hz	2.3207	0.034315	67.6301
Sinusoidal 1 Hz	1.0068	0.025304	39.7895
Triangular 5 Hz	0.65835	0.056952	11.5597
Sinusoidal 5 Hz	0.77694	0.019177	40.5145
**Reference**	**RMSE**		
	**PID**	**ANN-BackStepping**	**Difference**
Triangular 1 Hz	0.68476	0.040637	16.8507
Sinusoidal 1 Hz	0.35503	0.022215	15.9817
Triangular 5 Hz	1.1929	0.20345	5.8636
Sinusoidal 5 Hz	1.4060	0.036905	38.0979
**Reference**	**RRMSE**		
	**PID**	**ANN-BackStepping**	**Difference**
Triangular 1 Hz	0.0013979	0.00020317	6.8803
Sinusoidal 1 Hz	0.00072477	0.00011107	6.5255
Triangular 5 Hz	0.013333	0.0022739	5.8636
Sinusoidal 5 Hz	0.015715	0.00041249	38.0979
**Reference**	**IAU**		
	**PID**	**ANN-BackStepping**	**Difference**
Triangular 1 Hz	62.2480	61.7750	1.0076
Sinusoidal 1 Hz	63.3267	63.0855	1.0038
Triangular 5 Hz	66.9143	62.1487	1.0766
Sinusoidal 5 Hz	68.1085	65.7737	1.03549

## Data Availability

The raw data supporting the conclusions of this article will be made available by the authors on request.

## Abbreviations

The following abbreviations are used in this manuscript:

PEA	Piezoelectric actuator
ANN	Artificial Neural Network
J-A	Jiles–Atherton
P-I	Prandtl–Ishlinskii
RTI	Real-Time Interface
TDNN	Time Delay Neural Network
FF	Feed Forward
PID	Proportional Integral Derivative
IAE	Integral Absolute Error
RMSE	Root Mean Square Error
RRMSE	Relative Root Mean Square Error
